# Tracheostomy practice in a uk dgh: are we moving with the times?

**DOI:** 10.1186/2197-425X-3-S1-A932

**Published:** 2015-10-01

**Authors:** F Donaldson, C Mead, A Cormack, T Szakmany

**Affiliations:** Royal Glamorgan Hospital, Llantrisant, United Kingdom

## Introduction

Tracheostomy is commonly performed in critically ill patients with the objective of increasing comfort and shortening the duration of sedation, mechanical ventilation, and intensive care stay [1]. As the percutaneous technique has become widely available, the earlier use of tracheostomy has become commonplace [2, 3]. Consequently, there is ongoing debate about the benefits of early tracheostomy [4].

## Objectives

To review all tracheostomies since 2009 in our ICU, to identify whether any changes in practice are evident and if so, are they affecting outcomes?

## Methods

Retrospective case review using our electronic clinical information system between 2009-2014. Inclusion criteria: Patients admitted to Royal Glamorgan Hospital 10 bedded Critical Care unit, who underwent tracheostomy placement during admission. We excluded laryngectomy patients. We collected data on patient characteristics, timing of tracheostomy, number of extubation attempts, time to wean to tracheostomy mask and mortality. For statistical analysis Mann-Whitney U and Chi-square test was used.

## Results

Patient age, gender distribution and APACHE II scores were similar during the observation period. There was a significant reduction in the number of procedures compared to 2009 and there was also a clear trend for performing tracheostomies later from 2012. In 2014 there was a dramatic reduction in the number of procedures, with apparently increased mortality. However, overall outcome of the patients ventilated for >5 days with or without tracheostomies has been unchanged during the years. In 2014 none of the tracheostomies were performed to enable the reduction of sedation and all of them were preceded by at least one attempt for extubation.

## Conclusions

There has been a marked change in the clinical practice on our unit, especially after the publication of the TracMan trial, with evidence of tracheostomies performed later and in a more selected patient population [5]. Based on these results we now require that a tracheostomy for non-airway reasons is based on a multidisciplinary decision, preceded by at least two extubation attempts and done not earlier than day 10 of mechanical ventilation.

**Table 1:** summarises the main findings. *p < 0.05

	2009	2010	2011	2012	2013	2014
Total number of Critical care admissions	626	633	551	542	565	593
Number of patients ventilated for >5 days	71	82	73	81	84	60
Total number of tracheostomies	55	66	34	36	38	18*
Number of days ventilated before tracheostomy (median)	4	5	5	6	7	9*
Number of days from tracheostomy to tracheostomy mask (median)	6	7	10	7	6	9
Number of extubation attempts before tracheostomy (median)	0	0	0	0	1	2*
Mortality of patients with tracheostomy	22%	20%	15%	14%	11%	33%
